# Dissociation Between Whole-Blood and Kidney Tissue Tacrolimus Exposure in Kidney Transplant Recipients

**DOI:** 10.3390/jox16060196

**Published:** 2026-10-14

**Authors:** Sho Nishida, Natsuka Kimura, Naoki Sano, Tamaki Miura, Takehiro Ohyama, Kyoko Minamisono, Tamaki Ishima, Toshimasa Nakao, Noriyoshi Fukushima, Ryozo Nagai, Daiki Iwami, Kenichi Aizawa

**Affiliations:** 1 Department of Translational Research, Clinical Research Center, Jichi Medical University Hospital, Shimotsuke 329-0498, Japan; 2 Division of Renal Surgery and Transplantation, Department of Urology, Jichi Medical University, Shimotsuke 329-0498, Japan; 3 Department of Pathology, Jichi Medical University, Shimotsuke 329-0498, Japan; 4 Jichi Medical University, Shimotsuke 329-0498, Japan; 5 Clinical Pharmacology Center, Jichi Medical University Hospital, Shimotsuke 329-0498, Japan

**Keywords:** kidney transplantation, tacrolimus, therapeutic drug monitoring, kidney tissue, tissue-level drug exposure, tacrolimus nephrotoxicity, liquid chromatography–tandem mass spectrometry, precision medicine

## Abstract

Tacrolimus (TAC) therapeutic drug monitoring (TDM) routinely monitors whole-blood TAC concentrations; however, these measurements may not accurately reflect renal TAC exposure. We investigated the relationship between whole-blood and kidney tissue TAC exposure in kidney transplant recipients receiving long-term TAC therapy. Thirty-five kidney transplant recipients who underwent graft biopsy and TDM, including trough concentration (C_0_) and 24-h area under the concentration–time curve (AUC_0–24_), were enrolled from 2020–2021. TAC concentrations in kidney tissue (C_T_) were quantified using liquid chromatography–tandem mass spectrometry. Recipients were stratified according to their alternative arteriolar hyalinosis (aah) scores (Banff classification) into a Low-aah group (aah 0–1, n = 20) and a High-aah group (aah 2–3, n = 15). Whole-blood TAC concentrations, C_T_, and kidney-to-blood TAC concentration ratios (KBRs) were compared. KBRs were calculated using TAC concentrations measured at trough (C_0_), biopsy-matched (C_matched_), and peak (C_peak_) time points. Associations between time since transplantation and KBRs were subsequently assessed. Whole-blood TAC concentrations, C_T_, and KBRs were comparable between the two groups. However, a positive relationship between KBRs and time since transplantation was observed in the High-aah group, whereas no such pattern was observed in the Low-aah group. These exploratory findings suggest that the relationship between whole-blood and kidney tissue TAC exposure may differ according to time since transplantation, particularly in recipients with severe arteriolar hyalinosis. Tissue-based assessment of TAC exposure may provide pharmacokinetic information complementary to that from conventional whole-blood monitoring, offering a framework for future mechanistic investigation of TAC nephrotoxicity.

## 1. Introduction

Tacrolimus (TAC), a calcineurin inhibitor originally isolated from *Streptomyces tsukubaensis*, is the cornerstone immunosuppressive agent in kidney transplantation [1]. TAC suppresses T-cell activation primarily through inhibition of the calcineurin signaling pathway [2]. TAC has markedly improved short-term graft survival after kidney transplantation. Consequently, TAC-based immunosuppressive regimens are currently used in approximately 90% of kidney transplant recipients worldwide [3]. Despite these advances, however, long-term graft survival has improved only modestly. One major contributor to suboptimal long-term graft outcomes is tacrolimus-induced chronic nephrotoxicity (TAC nephrotoxicity), an unavoidable complication of prolonged TAC therapy [4,5].

TAC nephrotoxicity is a progressive and largely irreversible form of kidney injury caused by prolonged exposure to TAC. Histopathological hallmarks of this nephrotoxicity include characteristic arteriolar hyalinosis and tubulointerstitial fibrosis [4,5]. Histological features consistent with TAC nephrotoxicity have been reported in approximately 70% of kidney allografts after 10 years of TAC exposure [6]. At present, the definitive diagnosis of TAC nephrotoxicity relies on kidney allograft biopsy. Consequently, TAC nephrotoxicity is often diagnosed only after irreversible structural injury has already developed [4,6]. Furthermore, mechanisms underlying disease progression remain incompletely understood, and no established strategy is currently available to prevent or reverse TAC nephrotoxicity. These limitations highlight the need for biomarkers that better reflect progression of TAC-induced tissue injury before irreversible histological damage becomes apparent.

TAC pharmacokinetics and pharmacological activity are influenced by multiple factors, including genetic polymorphisms and drug–drug interactions. Therefore, individualized therapeutic management is essential [1]. To maintain TAC exposure within its narrow therapeutic window, therapeutic drug monitoring (TDM), based primarily on trough concentrations and the area under the concentration–time curve (AUC), has become the standard approach for dose optimization [7]. Additional clinical strategies, including TAC dose reduction, use of alternative immunosuppressive agents, and TAC withdrawal, have also been investigated to minimize nephrotoxicity [8,9,10,11]. Nevertheless, these strategies have not substantially improved long-term graft survival, suggesting that current whole-blood TDM alone may be insufficient to fully characterize renal TAC exposure. In current clinical practice, TAC remains indispensable for preventing rejection, despite its well-recognized nephrotoxicity.

In recent years, increasing attention has been directed toward measuring TAC concentrations in target cells and tissues, in addition to whole-blood concentrations, to optimize immunosuppressive therapy [12,13]. Among these approaches, quantifying TAC concentrations in kidney tissue may provide a more direct assessment of local TAC exposure at the primary site of TAC-induced injury [12]. However, it remains unclear whether whole-blood TAC concentrations accurately reflect kidney tissue TAC exposure during prolonged TAC therapy, much less how this relationship changes with severity of TAC nephrotoxicity. Therefore, we hypothesized that the relationship between whole-blood and kidney tissue TAC concentrations differs according to the duration of TAC exposure, particularly in recipients with severe arteriolar hyalinosis.

## 2. Materials and Methods

### 2.1. Patients

**Figure 1:**
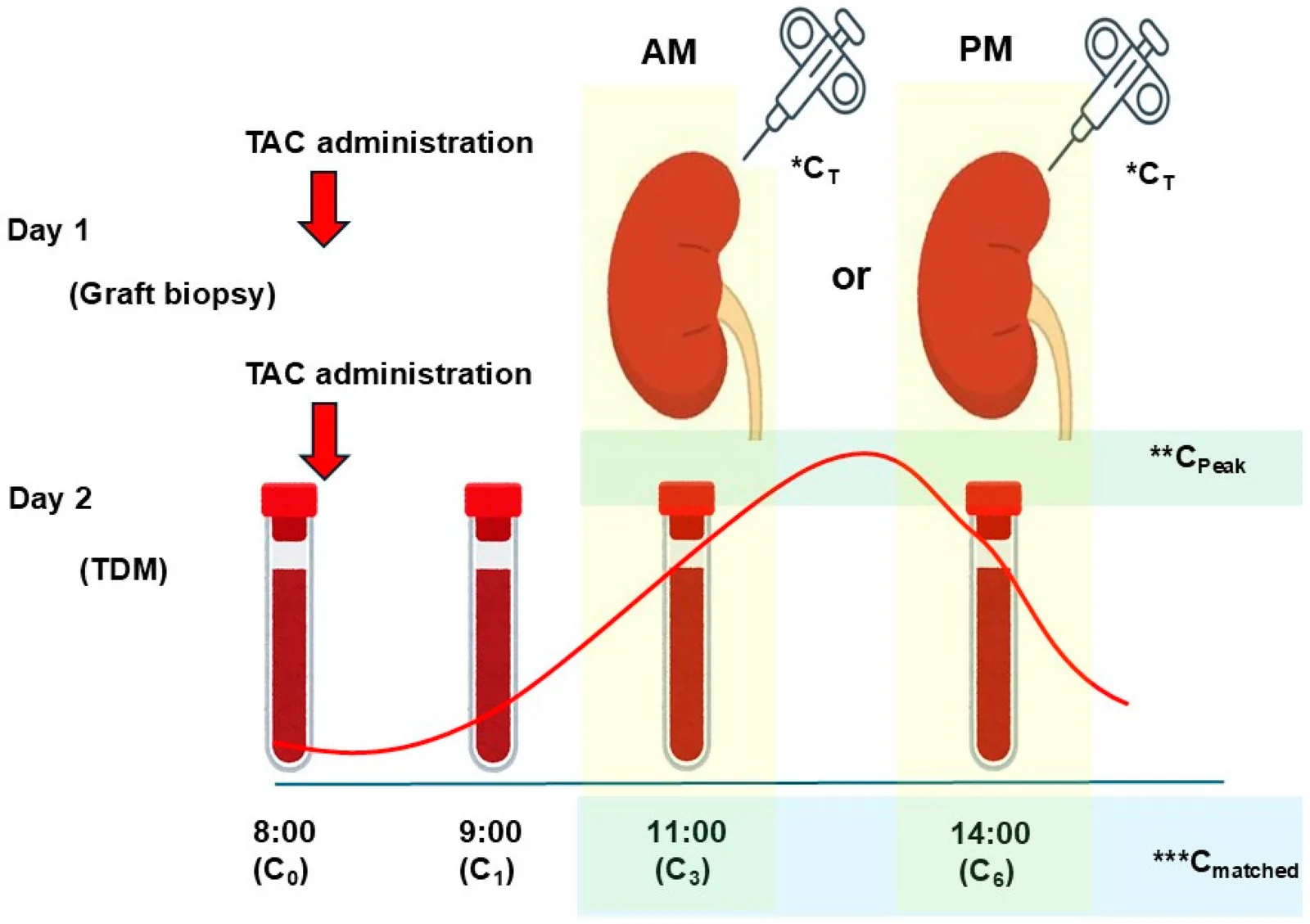
Sample collection protocol for kidney tissue and whole-blood TAC concentration measurements. All procedures were performed during hospitalization. Kidney allograft biopsy was performed on Day 1, either in the morning (approximately 11:00–12:00) or in the afternoon (approximately 13:00–14:00). Multiple blood samples for therapeutic drug monitoring were collected on Day 2 at trough (C_0_), and 1, 3, and 6 h after TAC administration (C_1_, C_3_, and C_6_, respectively). The red line schematically represents the time course of whole-blood TAC concentrations. *C_T_ represents the TAC concentration in kidney tissue. **C_peak_ was defined as the highest whole-blood TAC concentration among C_0_–C_6_. ***C_matched_ represents the whole-blood TAC concentration corresponding to the biopsy time (morning or afternoon).

This retrospective observational study included 35 consecutive kidney transplant recipients who underwent kidney graft biopsy and TDM of TAC between September 2020 and September 2021. All recipients had received once-daily extended-release tacrolimus (TAC-ER) for at least 1 year after kidney transplantation. Kidney allograft biopsy was performed on the first day of hospitalization, followed by multiple blood sampling for TDM on the second day (Figure 1). According to medical records, TAC dose and administration time remained unchanged, and no clinically relevant change in patient condition was documented between the kidney biopsy and pharmacokinetic sampling days. Alternate qualitative scoring for hyaline arteriolar thickening (aah scoring) was independently reviewed by two pathologists who were blinded to clinical information. Discordant cases were reviewed jointly, and final consensus scores were assigned [5,14].

Based on the revised aah score, recipients were classified into two groups: a Low-aah group (aah 0–1, n = 20) and a High-aah group (aah 2–3, n = 15). Clinical characteristics, whole-blood TAC concentrations, kidney tissue TAC concentrations, and kidney-to-blood TAC concentration ratios (KBRs) were compared between the two groups. KBRs were calculated by dividing kidney tissue TAC concentration (C_T_) by the corresponding whole-blood TAC concentration (C_T_/C_0_, C_T_/C_matched_, and C_T_/C_peak_). Because kidney biopsy and blood sampling were performed on consecutive days, C_matched_ was defined as the whole-blood TAC concentration corresponding to the biopsy time (morning or afternoon). C_peak_ was defined as the highest whole-blood TAC concentration obtained during pharmacokinetic sampling (C_0_–C_6_). Correlations between time since transplantation and C_T_ or KBRs were subsequently evaluated in each group.

### 2.2. Whole-Blood Sampling and TAC Concentration Measurement

Whole-blood TAC concentrations were measured using an electrochemiluminescence immunoassay, as previously described [15]. Blood collection time points were as follows: C_0_: trough, C_1_, 1 h after TAC administration; C_3_, 3 h after TAC administration; C_6_, 6 h after TAC administration. AUC was estimated using the following equation, as reported previously [16]:

AUC_0–24_ = 0.28 + 11.85 × C_0_ + 1.72 × C_1_ + 2.88 × C_3_ + 7.17 × C_6_

### 2.3. Kidney Tissue Collection and TAC Quantification

#### 2.3.1. Kidney Tissue Sampling

Kidney allograft biopsies were performed under ultrasound guidance with local anesthesia. Needle biopsy specimens were obtained from the renal cortex. Immediately after collection, tissue samples were snap-frozen and stored at −80 °C until analysis.

#### 2.3.2. Chemicals and Reagents

Tacrolimus (FK-506) was purchased from Cayman Chemical (Ann Arbor, MI, USA). Internal standard (DOSIMMUNE internal standard including ^13^C,^2^H_4_-TAC) was purchased from Shimadzu (Kyoto, Japan; manufactured by Alsachim, Illkirch-Graffenstaden, France). A 0.1 M zinc sulfate solution was purchased from Nacalai Tesque (Tokyo, Japan). Normal human control whole-blood samples (with EDTA-2K added) were purchased from BizCom Japan, Inc. (Tokyo, Japan). All other solvents were purchased from FUJIFILM Wako Pure Chemical Corporation (Osaka, Japan).

#### 2.3.3. Preparation of Calibration Samples and Tissue Samples

Tacrolimus was dissolved in dimethyl sulfoxide (DMSO) to a concentration of 5 mg/mL. The tacrolimus solution was diluted with methanol and added to human whole blood at a concentration of 50 ng/mL to prepare the highest calibration standard. That standard was then serially diluted with human whole blood to create calibration samples ranging from 0.05 to 50 ng/mL. Aliquots (50 μL) of calibration samples were placed in 1.5-mL tubes and vortexed (30 s) after adding 10 μL of internal standard, 80 μL of extraction buffer (MeOH/0.1 M zinc sulfate solution = 70/30) and 20 μL of MeOH. After centrifugation (12,000 rpm, 10 min, 4 °C), 110 μL of supernatant was transferred into vials for LC–MS/MS analysis.

Kidney samples were weighed before freezing and then placed in 2-mL hard tubes and homogenized (6.5 m/s, 5 s, 3 times) after adding 10 μL of internal standard, 80 μL of extraction buffer (MeOH/0.1 M zinc sulfate solution = 70/30), 20 μL of MeOH and 5-mm stainless steel beads. After centrifugation (12,000 rpm, 10 min, 4 °C), 110 μL of supernatant was transferred to vials for LC–MS/MS analysis.

#### 2.3.4. LC–MS/MS Analysis

Tacrolimus concentrations in kidney tissue were analyzed using an LC–MS/MS system (LCMS-8050, Shimadzu, Kyoto, Japan). For liquid chromatography (LC) analyses, a DOSIMMUNE Analytical column (Shimadzu) was utilized, with the column oven maintained at 50 °C and the autosampler set to 4 °C. Mobile phase A consisted of 90% 3 mM ammonium formate in water and 10% methanol, and mobile phase B contained 3 mM ammonium formate in methanol. The flow rate was set to 0.3 mL/min. The gradient program was as follows: 0–1 min, %B = 10; 1–4 min, %B = 10–80; 4–6 min, %B = 80–95; 6.1–6.3 min, %B = 100; 6.31 min, %B = 10. TAC was detected in electrospray ionization-positive mode. MS/MS conditions were as follows: nebulizer gas flow, 3 L/min; heating gas flow, 10 L/min; interface temperature, 300 °C; desolvation line temperature, 526 °C; heat block temperature, 400 °C; and drying gas flow, 10 L/min. TAC and its internal standard (^13^C,^2^H_4_-TAC) were quantified using multiple reaction monitoring transitions of 821.5 > 768.25 (TAC) and 826.5 > 773.4 (^13^C,^2^H_4_-TAC), respectively, in positive-ESI mode. Collision energies for each analyte were −23 V for TAC and −23 V for ^13^C,^2^H_4_-TAC. Sample concentrations were quantified by calculating the area ratio relative to the internal standard reagent added to each sample.

### 2.4. Statistical Analysis

Statistical analyses were performed using GraphPad Prism version 7.04 (GraphPad Software, Boston, MA, USA) and EZR (Saitama Medical Center, Jichi Medical University, Saitama, Japan), a graphical user interface for R (R version 4.2.2; accessed 4 October 2026). Continuous variables were compared using the Mann–Whitney U test. Categorical variables were analyzed using the chi-square test. Pearson’s correlation coefficient was used for the primary correlation analyses. To account for multiple comparisons across the eight correlation tests, Bonferroni-adjusted *p* values were calculated. Spearman’s rank correlation was additionally performed as a sensitivity analysis. Leave-one-out sensitivity analyses were performed in the High-aah group to assess the influence of individual observations on correlation estimates. Additional multiple linear regression analyses were performed to assess the interaction between time since transplantation and aah group, to evaluate potential confounding by biopsy timing, and to address potential limitations of ratio-based analyses. For the latter, kidney tissue TAC concentration (C_T_) was used as the dependent variable, with the corresponding whole-blood TAC concentration and time since transplantation included as independent variables.

## 3. Results

### 3.1. Patient Characteristics and Whole-Blood TAC Concentrations

Patient characteristics were comparable between the Low- and High-aah groups, with no significant differences observed in baseline demographic or clinical variables. Biopsy timing distribution did not differ significantly between the Low- and High-aah groups (Table 1). Whole-blood TAC pharmacokinetic parameters, including trough concentration (C_0_), post-dose concentrations (C_1_, C_3_, and C_6_), and AUC_0–24_, also did not differ significantly between the two groups (Table 2).

### 3.2. Kidney-Tissue TAC Concentration and Kidney-to-Blood TAC Concentration Ratios

**Figure 2:**
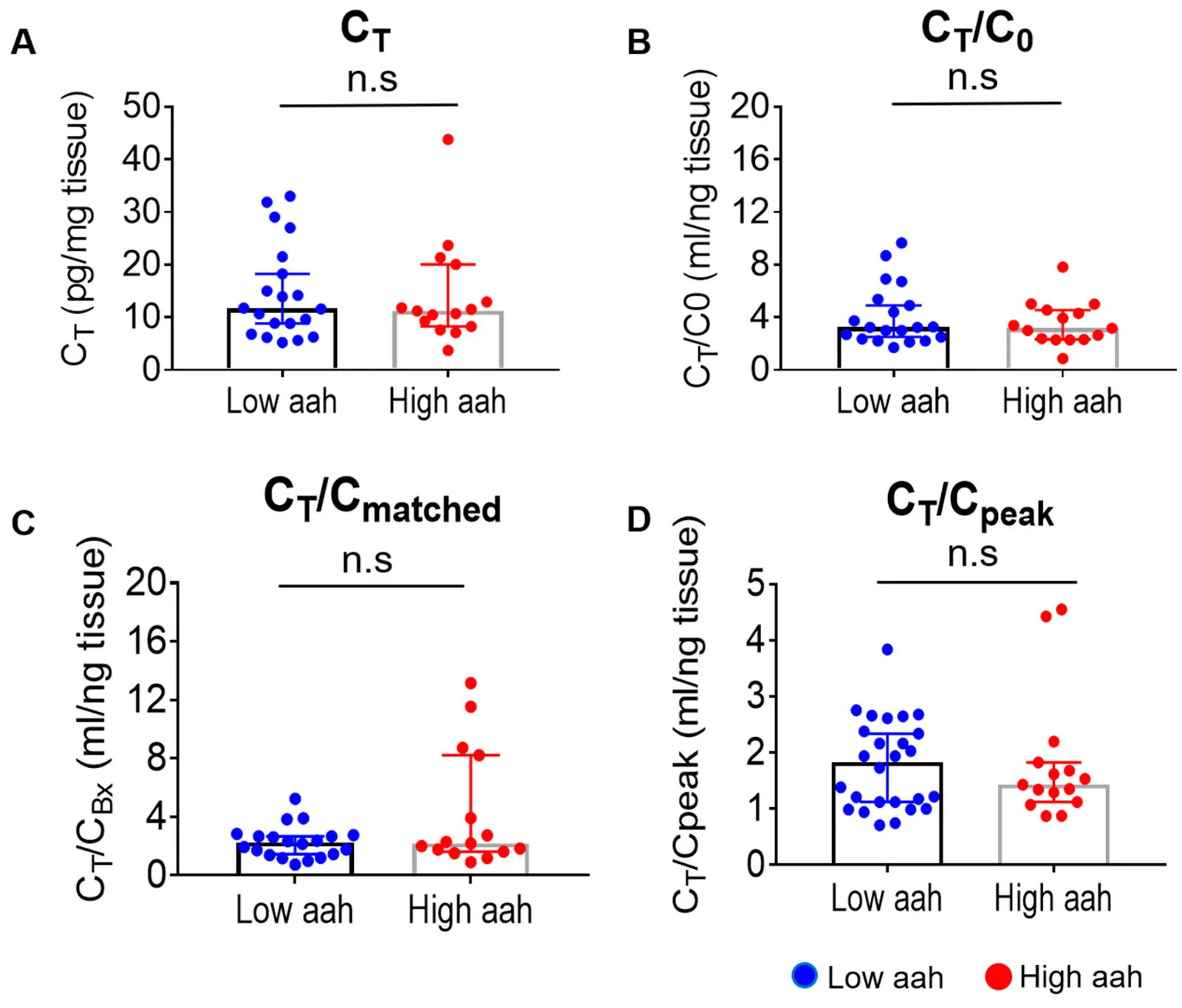
Comparison of kidney tissue TAC concentration and kidney-to-blood TAC concentration ratios (KBRs) between the Low- and High-aah groups. (**A**) Kidney tissue TAC concentration (C_T_). (**B**–**D**) KBRs calculated as C_T_/C_0_, C_T_/C_matched_, and C_T_/C_peak_, respectively. Data are presented as individual values with the median. Statistical comparisons were performed using the Mann–Whitney U test. n.s indicates “not significant.” Abbreviations: C_T_, TAC concentration in kidney tissue; C_matched_, whole-blood TAC concentration corresponding to the biopsy time; C_peak_, highest whole-blood TAC concentration measured during pharmacokinetic sampling (C_0_–C_6_); KBRs, kidney-to-blood TAC concentration ratios.

Absolute TAC concentrations in kidney graft tissue (C_T_) were comparable between the Low- and High-aah groups (Figure 2A). Similarly, KBRs showed no significant differences between the two groups (Figure 2B–D).

### 3.3. Association Between Time Since Transplantation and Kidney-to-Blood TAC Concentration Ratios

**Figure 3:**
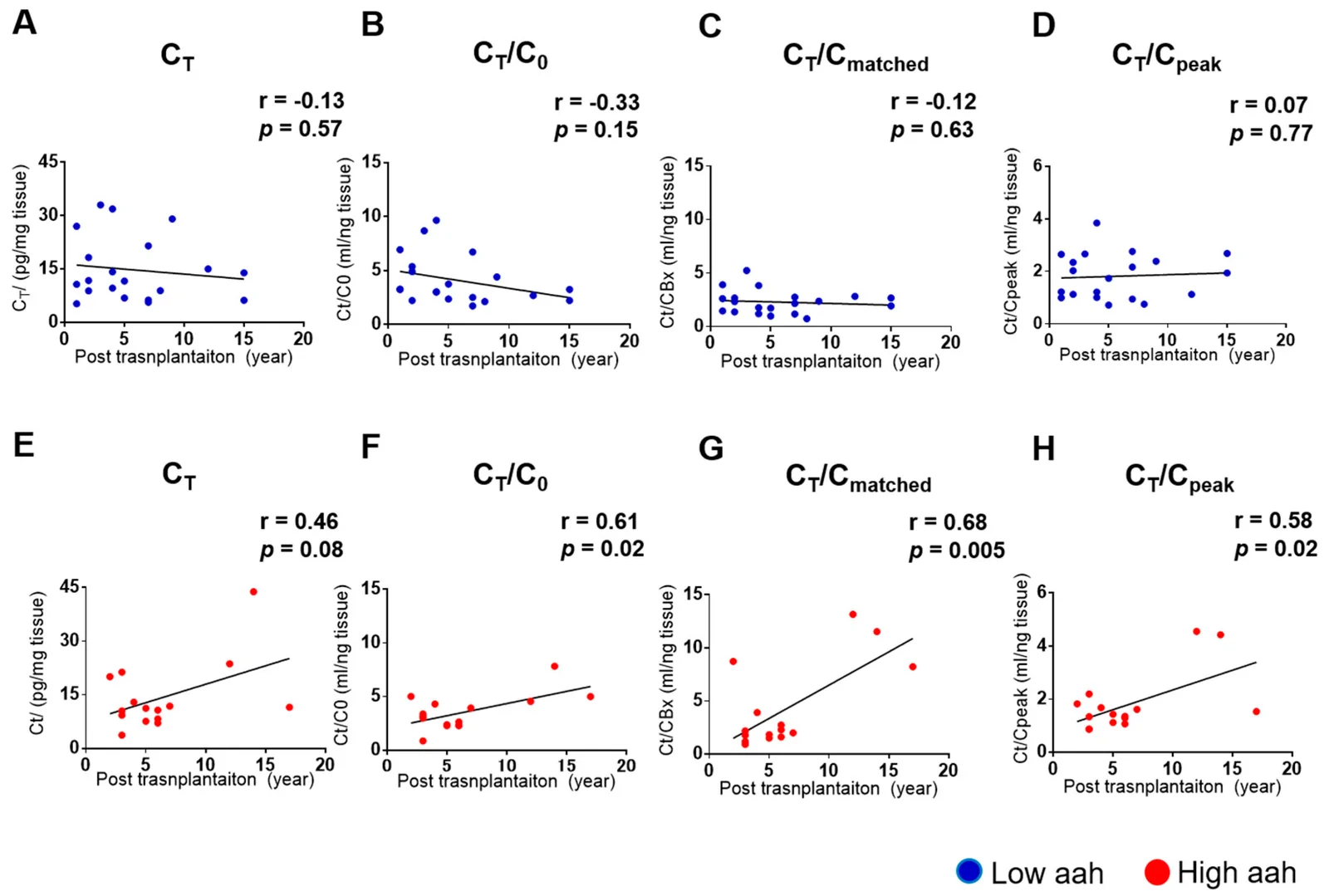
Association between time since transplantation and kidney tissue TAC concentration and kidney-to-blood TAC concentration ratios (KBRs). (**A**–**D**) Correlations between time since transplantation and KBRs in the Low-aah group. (**E**–**H**) Corresponding analyses in the High-aah group. Pearson’s correlation coefficient and simple linear regression analysis were used to evaluate associations. *r* indicates the Pearson correlation coefficient. *p* values shown in the figure are unadjusted. To account for multiple comparisons across the eight correlation analyses, Bonferroni-adjusted *p* values were also calculated. Correlation coefficients with 95% confidence intervals, unadjusted and Bonferroni-adjusted *p* values, and sensitivity analyses are provided in Supplementary Table S1. Spearman’s rank correlation was also performed as a sensitivity analysis, with detailed results provided in Supplementary Table S1. Abbreviations: C_T_, TAC concentration in kidney tissue; C_matched_, whole-blood TAC concentration corresponding to biopsy timing; C_peak_, highest whole-blood TAC concentration measured during pharmacokinetic sampling (C_0_–C_6_); KBRs, kidney-to-blood TAC concentration ratios.

The relationship between time since transplantation and kidney tissue TAC exposure was subsequently evaluated. No significant correlation was observed between time since transplantation and absolute kidney tissue TAC concentration (C_T_) in either group (Figure 3A,E). In the High-aah group, longer time since transplantation was positively correlated with C_T_/C_0_ (r = 0.608, 95% CI 0.138–0.854, *p* = 0.016), C_T_/C_matched_ (r = 0.687, 95% CI 0.270–0.887, *p* = 0.0046), and C_T_/C_peak_ (r = 0.580, 95% CI 0.096–0.842, *p* = 0.024) (Figure 3F–H). In contrast, no significant correlations between time since transplantation and KBRs were observed in the Low-aah group (Figure 3B–D; Supplementary Table S1). Formal interaction analyses showed significant interactions between time since transplantation and aah group for C_T_/C_0_ (B = 0.401, 95% CI 0.092–0.711, *p* = 0.013) and C_T_/C_matched_ (B = 0.656, 95% CI 0.304–1.008, *p* < 0.001), but not for C_T_/C_peak_ (B = 0.135, 95% CI −0.015–0.285, *p* = 0.076) (Supplementary Table S2). After Bonferroni correction for the eight correlation tests, the association with C_T_/C_matched_ remained statistically significant (adjusted *p* = 0.037), whereas associations with C_T_/C_0_ (adjusted *p* = 0.130) and C_T_/C_peak_ (adjusted *p* = 0.188) did not. In sensitivity analyses, Spearman’s rank correlations showed positive directions for all three KBR measures in the High-aah group, although none reached statistical significance. Leave-one-out analyses showed consistently positive correlations across all iterations, with C_T_/C_matched_ demonstrating the greatest robustness to exclusion of individual observations (Supplementary Table S1). Time since transplantation was compared according to biopsy timing and did not significantly differ between morning and afternoon biopsies. Furthermore, in the High-aah group, the associations between time since transplantation and KBRs remained statistically significant after adjustment for biopsy timing (Supplementary Table S2). We also performed multiple linear regression analyses using C_T_ as the dependent variable and the corresponding whole-blood TAC concentration and time since transplantation as separate predictors. In the High-aah group, time since transplantation showed a positive regression coefficient in all three models. The association was statistically significant after adjustment for C_0_ and C_peak_, whereas it did not reach statistical significance after adjustment for C_matched_ (Supplementary Table S2).

## 4. Discussion

The principal finding of this study is that longer time since transplantation was associated with higher kidney-to-blood TAC concentration ratios in recipients with severe arteriolar hyalinosis. Notably, this discrepancy between whole-blood and tissue-level TAC exposure was observed despite comparable whole-blood TAC concentrations and absolute tissue TAC concentrations between the groups. To our knowledge, this is the first cross-sectional study to show an association between longer time since transplantation and higher kidney-to-blood TAC concentration ratios in recipients with High-aah scores.

A major strength of the present study is rigorous histopathological evaluation. The aah score was proposed as an alternative pathological parameter to provide a more specific and reproducible assessment of TAC nephrotoxicity than the ah score [5]. The aah score is graded from 0 to 3 according to the severity of arteriolar hyalinosis and was used in the present study as a histopathological marker associated with TAC nephrotoxicity [5,14,17]. Histological severity was reassessed by blinded pathologists using the aah score, thereby minimizing potential bias introduced by clinical information and allowing objective stratification according to the severity of arteriolar hyalinosis.

Despite objective histological stratification, baseline characteristics and whole-blood TAC concentrations were comparable between the two groups. Current clinical guidelines recommend TDM based on whole-blood TAC concentrations, and all recipients in the present study were managed according to these recommendations [18]. Nevertheless, recipients with severe arteriolar hyalinosis were identified, suggesting that whole-blood TAC concentrations alone may not adequately reflect tissue-level TAC exposure or the pharmacological burden in the kidney graft.

One possible explanation is the unique pharmacokinetic profile of tacrolimus. Approximately 90% of circulating TAC resides in erythrocytes, such that only a small unbound fraction is considered pharmacologically active [19]. Consequently, whole-blood TAC concentrations may not represent the amount of TAC reaching kidney tissue or exerting biological effects in the graft. Increasing KBRs observed in recipients in the High-aah group support the possibility that the relationship between tissue-level and systemic TAC exposure may differ according to time since transplantation.

Previous studies have quantified TAC concentrations in tissue and have compared them with whole-blood TAC concentrations. However, the reported relationship between blood and tissue TAC concentrations has been inconsistent [13,20]. In the present study, absolute TAC concentration in kidney tissue was also comparable between the two groups (Figure 2A). One possible explanation is that tissue TAC concentration is influenced by biopsy timing relative to TAC administration, which is difficult to standardize in routine clinical practice. Therefore, absolute tissue TAC concentration measured at a single biopsy may not adequately reflect overall tissue-level TAC exposure or disease severity.

To minimize the influence of biopsy timing, we evaluated KBRs, which normalize tissue TAC concentration to corresponding whole-blood TAC concentrations. Although KBRs were comparable between the Low- and High-aah groups in the cross-sectional comparison (Figure 2B–D), a different pattern emerged when time since transplantation was taken into account. In the High-aah group, longer time since transplantation showed positive associations with all three KBR measures (Figure 3F–H), whereas no significant associations were observed in the Low-aah group (Figure 3B–D). However, after correction for multiple comparisons, only the association with C_T_/C_matched_ remained statistically significant. Although the positive direction of these associations was generally maintained in sensitivity analyses, their statistical significance varied depending on the analytical approach. In additional analyses that modeled tissue TAC concentration directly rather than as a ratio, time since transplantation also showed positive regression coefficients in all three models after accounting for the corresponding whole-blood TAC concentration in the High-aah group, although statistical significance was not observed in the C_matched_-adjusted model. Interaction analyses further showed significant interactions between time since transplantation and aah group for C_T_/C_0_ and C_T_/C_matched_, but not for C_T_/C_peak_. Therefore, these findings should be considered exploratory and hypothesis-generating. Nevertheless, the generally positive direction of these associations across different analytical approaches raises the possibility that the relationship between systemic and tissue-level TAC exposure may differ according to time since transplantation.

Biological mechanisms underlying this dissociation remain unclear. However, several pharmacokinetic and tissue-specific factors may contribute to this phenomenon. Prolonged TAC exposure reportedly induces cellular injury and progressive fibrosis in experimental and clinical settings [21,22,23,24]. In addition, patients with TAC-associated acute kidney injury exhibit disproportionately higher TAC concentrations in kidney tissue relative to whole-blood concentrations [12].

Several studies have also highlighted the clinical importance of intrapatient variability (IPV) in whole-blood TAC concentrations. Increased IPV has consistently been associated with inferior graft outcomes [25,26]. In addition, prolonged exposure to supratherapeutic TAC concentrations in recipients with high IPV further increases the risk of TAC nephrotoxicity [27]. Although previous studies have focused on temporal variability of whole-blood TAC concentrations, the relationship between whole-blood and kidney tissue TAC exposure remains poorly understood. Our findings suggest that blood–tissue dissociation may represent an additional dimension of interindividual variability in TAC exposure. Although C_0_ remains the most widely used parameter for TAC TDM, AUC- and concentration-to-dose ratio (C/D ratio)-based approaches may provide additional information regarding TAC exposure and pharmacokinetic variability [7,28]. Our findings suggest that tissue-based assessment of TAC exposure may provide complementary information beyond established blood-based TAC monitoring strategies, including C_0_, AUC, IPV and the C/D ratio. Given the invasive nature of kidney biopsy, further studies are needed to determine whether tissue TAC assessment provides additional clinical value in addition to these established monitoring approaches, including whether it improves TAC dosing, diagnosis or patient outcomes. Recent in vivo studies of chronic TAC nephrotoxicity have identified molecular alterations in kidney tissue and signaling pathways that may contribute to TAC-induced kidney injury [21,29,30,31]. However, validation of these molecular alterations in human kidney graft tissue remains limited. Recognition of dissociation between whole-blood and tissue-level TAC exposure may enable more refined stratification of TAC nephrotoxicity. Such stratification could facilitate human tissue-based validation of molecular alterations, ultimately contributing to biomarker discovery and identification of therapeutic targets. By integrating tissue-level TAC exposure with histological and molecular profiling, future studies may bridge experimental findings with clinical pathology, thereby advancing precision stratification and individualized management of TAC-induced chronic nephrotoxicity.

This study had several limitations. First, it was a single-center study with a relatively small sample size, which limited statistical power, and the present findings should therefore be considered exploratory and hypothesis-generating. Validation in larger multicenter cohorts is warranted. Second, kidney tissue and blood samples were not collected simultaneously because kidney biopsy and pharmacokinetic blood sampling were performed on consecutive days, although TAC dose and administration time remained unchanged, and no clinically relevant change in patient condition was documented between these assessments. In addition, longitudinal within-patient changes in tissue-level TAC exposure or the relationship between whole-blood and tissue TAC exposure could not be evaluated because serial kidney biopsies were not available. Advances in microsampling technologies may facilitate synchronized whole-blood pharmacokinetic assessment at the time of kidney biopsy, allowing more precise evaluation of tissue-level TAC exposure in future studies. Third, although TAC dose and CYP3A5 genotype were available, data on ABCB1 genotype, body weight, hematocrit, concomitant medications potentially affecting TAC metabolism, and detailed individual maintenance immunosuppressive regimens were not systematically available. Therefore, residual confounding by these pharmacokinetic and clinical factors cannot be excluded. Finally, histological severity may have been influenced by sampling variability inherent to kidney biopsy specimens. In addition, arteriolar hyalinosis and other histological findings associated with TAC nephrotoxicity are not entirely specific and may overlap with other forms of chronic allograft injury. Despite these limitations, the present study introduces the concept of dissociation in the relationship between whole-blood and tissue-level TAC exposure according to time since transplantation. These findings provide a new framework for understanding TAC nephrotoxicity and may facilitate future development of improved biomarkers and individualized therapeutic strategies.

## 5. Conclusions

The observed positive association between longer time since transplantation and KBRs may reflect a greater dissociation between whole-blood and kidney tissue TAC exposure in recipients with severe arteriolar hyalinosis. Although these findings are exploratory and require validation in larger cohorts, tissue-based assessment of TAC exposure may provide pharmacokinetic information complementary to conventional whole-blood monitoring and a framework for future mechanistic investigation of TAC nephrotoxicity.

## Figures and Tables

**Table 1:** Patient Clinical Characteristics.

		The Low-aah Group	The High-aah Group	*p*-Value
		n = 20	n = 15	
Gender	Male	8 (40%)	10 (66.7%)	0.12
Period of post kidney transplantation	years	4.5 (1–15)	5 (3–17)	0.60
Age	years old	47 (22–70)	42 (20–70)	0.32
Serum creatinine	mg/dL	1.13 (0.63–3.19)	1.23 (0.80–1.85)	0.62
Urine-protein	g/gCr	0.24 (0.00–14.6)	0.20 (0.00–1.89)	0.76
Episode of rejection	n (%)	3 (15)	1 (6.7)	0.44
Biopsy timing (morning)	n (%)	7 (35)	7 (46.7)	0.49
Tacrolimus dose	mg/day	3 (1–4)	2 (1.5–3)	0.67
CYP3A5*1/*3	n (%)	12 (60.0)	5 (33.3)	0.12
CYP3A5*3/*3	n (%)	8 (40.0)	10 (66.7)	

Data are presented as number (%) or median (range).

**Table 2:** Whole-blood and kidney-tissue TAC concentrations.

			The Low-aah Group	The High-aah Group	*p*-Value
			n = 20	n = 15	
Whole blood	C_0_	ng/mL	3.3 (1.2–6.6)	3.3 (2.1–6.3)	0.57
	C_1_	ng/mL	4.2 (2.4–8.1)	5.3 (2.2–11.0)	0.20
	C_3_	ng/mL	6.3 (2.9–12.4)	5.3 (1.8–9.9)	0.20
	C_6_	ng/mL	6.4 (2.0–13.4)	5.0 (1.4–10.0)	0.20
	AUC	ng/mL × hour	112.8 (56.3–195.0)	105.2 (76.4–178.3)	0.45
Kidney tissue	C_T_	pg/mg tissue	11.7 (6.3–33.0)	11.2 (8.3–43.8)	0.88

Data are presented as median (range).

## Data Availability

The data presented in this study are available on request from the corresponding author due to privacy and ethical restrictions.

## Supplementary Materials

The following supporting information can be downloaded at https://www.mdpi.com/article/10.3390/jox16060196/s1; Table S1: Correlation and sensitivity analyses of time since transplantation with kidney tissue tacrolimus concentration and kidney-to-blood tacrolimus concentration ratios; Table S2: Additional regression analyses of tacrolimus exposure in relation to time since transplantation.

## Abbreviations

The following abbreviations are used in this manuscript:

TAC	Tacrolimus
KBRs	Kidney-to-blood TAC concentration ratios
AUC	The area under the concentration–time curve
IPV	Intrapatient variability
TDM	Therapeutic drug monitoring
C_T_	Kidney tissue TAC concentration
C_matched_	Biopsy-matched TAC concentration
C_peak_	Peak TAC concentration
C/D ratio	Concentration-to-dose ratio
